# Compound C Protects Mice from HFD-Induced Obesity and Nonalcoholic Fatty Liver Disease

**DOI:** 10.1155/2019/3206587

**Published:** 2019-08-14

**Authors:** Fang Wang, Yuxing Liu, Jingjing Yuan, Wenjun Yang, Zhaohui Mo

**Affiliations:** ^1^The Endocrinology Department of the Third Xiangya Hospital, Central South University, Changsha, Hunan Province, China; ^2^The Life Science School of Medicine, Central South University, Changsha, Hunan Province, China

## Abstract

**Objectives:**

The aim of this study was to investigate the effects of compound C on an in vivo mouse model of high-fat diet- (HFD-) induced obesity and hepatosteatosis.

**Methods:**

C57BL/6 mice were fed with a standard diet (*n* = 5) for 16 weeks and then injected saline once a day for 4 weeks as the normal chow group. Mice (*n* = 10) were fed with HFD for 16 weeks to induce obesity and hepatosteatosis and then divided into two groups: HFD + vehicle group injected with the vehicle solution (saline) and HFD + compound C group injected with compound C in saline (5 mg/kg i.p., once a day) for 4 weeks. Liver histology was observed. The expression levels of genes related to lipid metabolism and proinflammation in liver tissue were examined. NLRP3 inflammasome expression in liver tissue was detected by the western blot assay. HepG2 cells were pretreated with compound C and/or AICAR for 1 h and then treated with palmitic acid (PA) for 3 h. The cells were collected, and mRNA levels were determined.

**Results:**

There was a significant reduction in body-weight gain and daily food intake in the HFD + compound C group compared with the HFD + vehicle group (*p* < 0.05). The glucose tolerance test (GTT) and insulin tolerance test (ITT) showed that compound C alleviated insulin resistance. Histology analysis showed a significant reduction of hepatic steatosis by compound C. Compound C also significantly decreased fatty acid synthesis genes, while increased fatty acid oxidation genes. Furthermore, compound C significantly reduced the expression of proinflammatory markers and NLRP3 inflammasome (*p* < 0.05). Compound C enhanced mRNA levels of SOD1, SOD2, catalase, GPx1, and GPx4 and reduced the p-AMPK/AMPK ratio, which were stimulated by palmitic acid (PA). The effect was enhanced by AICAR.

**Conclusion:**

Our data suggest that compound C is a potent NAFLD suppressor and an attractive therapeutic target for hepatic steatosis and related metabolic disorders.

## 1. Introduction

Nonalcoholic fatty liver disease (NAFLD), characterized by abnormal lipid accumulation in hepatocytes, is the global epidemic in one-third of the population in the world [1, 2]. NAFLD is commonly associated with metabolic comorbidities, such as visceral obesity, insulin resistance, hyperglycemia, and arterial hypertension [3]. Accordingly, NAFLD includes a wide spectrum of liver abnormalities ranging from simple steatosis to nonalcoholic steatohepatitis (NASH) and may potentially progress to advance liver diseases, cirrhosis, and hepatocellular carcinoma (HCC). However, currently, there are no approved pharmacologic agents for NAFLD treatment [4, 5]. Given the disease burden, it is critical and urgent to investigate the effective targets and develop the corresponding pharmacologic agents to treat NAFLD. In recent years, both basic and clinical research studies have established that sterile inflammation is a key driver in triggering the process from NAFLD to nonalcoholic steatohepatitis (NASH) [6, 7]. There is also compelling evidence that the nucleotide-binding domain of leucine-rich repeat protein 3 (NLRP3) inflammasome drives the sterile inflammation and plays a central role in the progression of NAFLD [8]. Moreover, the activation of NLRP3 inflammasome might lead to the NAFLD development and progression, including hepatic steatosis, liver injury, and fibrogenesis. Thus, NLRP3 inflammasome may serve as a promising new therapeutic target for the treatment of NAFLD/NASH [9].

**Figure 1:**
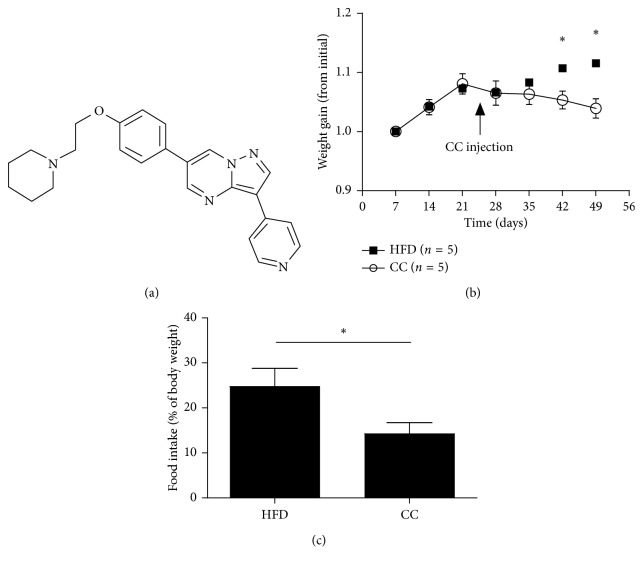
Compound C protects mice from HFD-induced obesity. (a) Structure of compound C. (b) Effect of compound C on body-weight gain of the mice (*n* = 5). (c) Effect of compound C on food intake of the mice. Results are presented as mean ± SEM. ^*∗*^*p* < 0.05, compared with the HFD group. HFD = high-fat diet; CC = compound C.

Compound C, 6-[4-(2-piperidin-1-yl-etoxy)-phenyl]-3-pyridin-4-yl-pyrazolo[1,5-a]pyrimidine, a part of a pyrazolopyrimidine class of protein kinase inhibitors (Figure 1(a)), has been described as a potent and highly selective inhibitor of AMP-activated protein kinase (AMPK) because it efficiently blocks metabolic actions of AMPK. Recently, contrary to expectations, compound C has been demonstrated to be not only with the AMPK inhibition effect but also with AMPK-independent pharmacological actions [10–12]. Reportedly, compound C significantly inhibits the adipogenic differentiation of 3T3-L1 cells to and enhances insulin sensitivity of adipocytes [13]. In our previous studies, we have unexpectedly found that compound C is an anti-inflammatory and anti-NLRP3 inflammasome agent in vitro. NLRP3 inflammasome plays an important role in NAFLD progression. It is reasonable to speculate that compound C might have therapeutic function in treatment of NAFLD in vivo.

In this study, we have investigated the effects of compound C on a mouse model of high-fat diet- (HFD-) induced obesity and hepatosteatosis. Interestingly, we have observed that compound C significantly reduces HFD-induced body-weight gain, insulin resistance, hepatic lipid accumulation, and inflammatory cytokine expression in the liver. In-depth investigations have shown that compound C suppresses NLRP3 inflammasome activation and improves fatty acid metabolism, leading to the protection from NAFLD development and metabolic complications.

## 2. Materials and Methods

### 2.1. Materials

Compound C was from Selleck Chemicals (Houston, TX, USA). Anti-NALP3, anti-AMPK*α*, anti-p-AMPK*α*, and anti-*β*-actin antibodies were from Cell Signaling Technology (Beverly, MA, USA). Anti-procaspase-1 and anti-ASC antibodies were from Abcam (Cambridge, UK).

### 2.2. Mouse NAFLD Model Establishment and Treatments

C57BL/6 mice at the age of 6 weeks (body weight = 23–25 g) (Slaccas Inc., Shanghai, China) were randomly assigned to three groups: mice in the normal chow (normal) group (*n* = 5) were fed with a standard diet for 16 weeks and then injected saline once a day for 4 weeks; 10 mice were fed with HFD (60 kcal% fat) for 16 weeks to induce obesity and hepatosteatosis and then divided into two groups: HFD + vehicle (HFD) group (*n* = 5) injected with the vehicle solution (saline) and HFD + compound C group (*n* = 5) injected with compound C in saline (5 mg/kg i.p., once a day) for 4 weeks.

We have investigated the suitable concentrations of compound C (1–10 mg/kg) for i.p. injection and found that 5 mg/kg of compound C was safe for daily injections for up to 30 days, which was consistent with that in the reported literatures. Control mice were intraperitoneally injected with the same volume of vehicle solution. All mice were weighed, and food intakes were measured weekly. The total weekly food intake of a mouse was calculated by measuring the food added subtracted by the food left in the cage divided by the number of mice in the cage. Mouse daily food intake was calculated by dividing total weekly food intake by 7. After completion of the treatment, mice were anesthetized and sacrificed by cardiac puncture. Thereafter, the livers were dissected. After dissection, a portion of fresh tissue was fixed in 10% buffered formalin, and the remaining specimens were immediately frozen in liquid nitrogen and then stored at −80°C. All animal protocols were approved by the Animal Care and Use Committee of the Third Xiangya Hospital of Central South University.

### 2.3. Glucose Tolerance Test (GTT) and Insulin Tolerance Test (ITT)

The GTT and ITT were performed in mice 19 weeks after HFD or NC feeding. For the GTT, mice were fasted overnight, followed by an intraperitoneal injection of 2 g/kg glucose. For the ITT, mice were fasted for 6 h, followed by an intraperitoneal injection of 0.75 units/kg insulin. Blood was obtained from the tail vein before (0 min) and after (15, 30, 60, 90 and 120 min) the injection of glucose or insulin injection. Glucose levels were measured using an automatic glucometer (Roche Diagnostics, Rotkreuz, Switzerland).

### 2.4. Histology

Liver samples fixed in 10% buffered formalin were embedded in paraffin, sliced (2 *μ*m sections), and stained with hematoxylin and eosin (H&E). Histological examination for morphological changes was performed in a blinded manner. Liver sections were scored according to the criteria of the NAFLD activity score (NAS).

### 2.5. Western Blot Analysis

For the western blot analyses, the total protein was extracted from liver tissue using the protein lysis buffer (KangChen Bio-tech, Shanghai, China). The protein concentrations were examined using BCA Protein Assay Reagent (Thermo Scientific, Rockford, USA). Cell lysates containing 30 *μ*g of total protein from different samples were subjected to 8% SDS-PAGE, followed by electrophoretic transfer to a PVDF membrane (Millipore). The membranes were blocked using 5% nonfat milk for 30 min and incubated with the primary antibodies specific for NLRP3 (1 : 1000), pro-caspase-1/cleaved caspase-1 (1 : 1000), ASC (1 : 1000), and *β*-actin or *α*-tubulin (1 : 10000) overnight at 4°C and then secondary antibodies at room temperature for 1 h. The membranes were detected using ECL Western Blotting Analysis System (Amersham). Band intensities were quantified using densitometry and ImageJ software, and the expression of target proteins was normalized to *β*-actin or *α*-tubulin. Western blotting was performed at least in duplicate for three independent experiments.

### 2.6. Cell Culture and Treatment

The human hepatoma HepG2 cells were cultured in a humidified atmosphere of 95% air plus 5% CO_2_ in a 37°C incubator in Dulbecco's modified Eagle's medium (DMEM) supplemented with 10% heat-inactivated fetal bovine serum (FBS). The cells were pretreated with 25 *μ*M of compound C, 25 *μ*M of compound C plus 500 *μ*M of 5-aminoimidazole-4-carboxamide-1-beta-D-ribofuranoside (AICAR), or the same volume of PBS for 1 h and then treated with 0.5 mM of palmitic acid (PA) for 3 h. The cells were collected for mRNA measurement.

### 2.7. Quantitative Real-Time PCR Analysis

Total RNA of the liver tissue or HepG2 cells was extracted by the TRIzol reagent (Invitrogen Life Technologies, Carlsbad, CA, USA) according to the manufacturer's protocol. A total of 2 *μ*g RNA was reverse transcribed into cDNA using PrimeScript™ RT Reagent Kit (Takara, Tokyo, Japan). PCR amplification was performed with SYBR Green PCR Master Mix. Quantitative real-time PCR of target cDNA was conducted for proinflammatory genes (IL-1*β*, IL-18, IL-6, and TNF-*α*), lipogenic genes (PPAR*γ*, FAS, and SREBP-1c), and fatty acid oxidation genes (PPAR*α*, LCAD, MCAD, SOD1, SOD2, catalase, GPx1, and GPx4). The target gene values were normalized to the values of *β*-actin and expressed as relative fold increase (2^−ΔΔCt^) over the control.

### 2.8. Statistical Analysis

The data were analyzed using SPSS 18.0 software (SPSS Inc, Chicago, IL, USA) and were presented as mean ± standard error (SEM). To examine the significant differences between groups, the data were analyzed using Student's *t*-test. *p* values <0.05 were considered statistically significant.

## 3. Results

### 3.1. Compound C Protects Mice from HFD-Induced Obesity and Insulin Resistance

**Figure 2:**
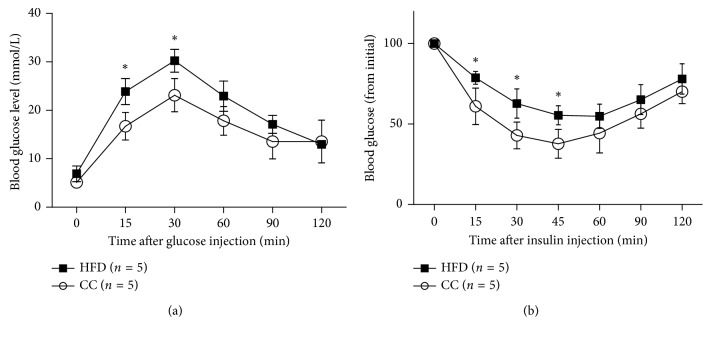
Compound C protects mice from HFD-induced insulin resistance. (a) Effect of compound C on the glucose tolerance test. (b) Effect of compound C on the insulin tolerance test. Results are presented as mean ± SEM. ^*∗*^*p* < 0.05, compared with the HFD group. HFD = high-fat diet; CC = compound C.

Since obesity is closely associated with NAFLD, we examined whether compound C influences the food intake and body-weight gain in mice. We compared the body-weight gain and food intake between the HFD group and the HFD plus compound C group. Compared with mice in the HFD group, there is a significant reduction in body-weight gain and daily food intake in mice in the compound C group (Figures 1(b) and 1(c)), which is consistent with that in the previous report, which suggests that the increased metabolic rate might provide a mechanism by which compound C protects mice from diet-induced obesity. Moreover, the glucose tolerance test and insulin tolerance test showed that the compound C-treated mice displayed the increase in the glucose and insulin tolerance when compared with HFD mice (Figures 2(a) and 2(b)). The data agree with previous findings that the compound C pretreatment reverses the impairment of AKT phosphorylation and ultimately enhances insulin sensitivity of adipocytes [13].

### 3.2. Compound C Alleviates HFD-Induced Hepatic Steatosis

**Figure 3:**
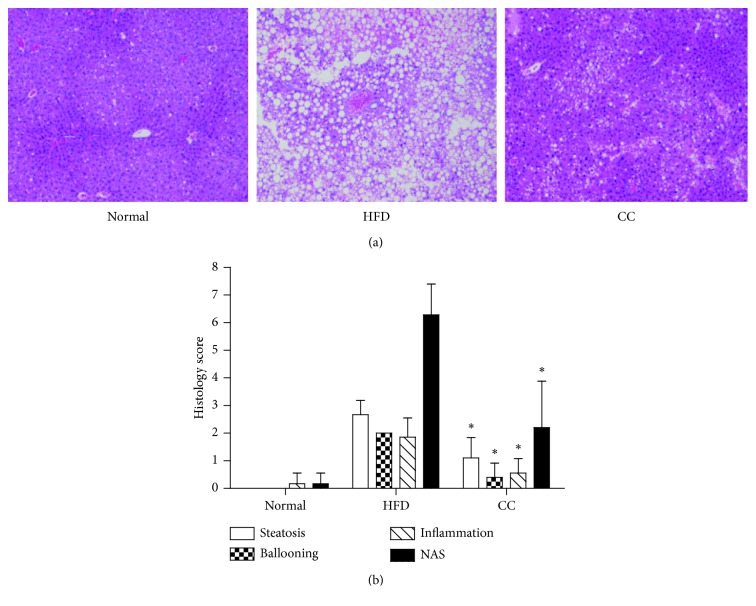
Representative microphotograph of hematoxylin and eosin staining of the hepatic lipid accumulation (normal, HFD, and HFD + compound C (5 mg/kg i.p., once a day) groups) and effects of compound C on hepatic steatosis in HFD-fed mice (a, b). ^*∗*^*p* < 0.05, compared with the HFD group (*n* = 5/group). HFD = high-fat diet; CC = compound C.

**Figure 4:**
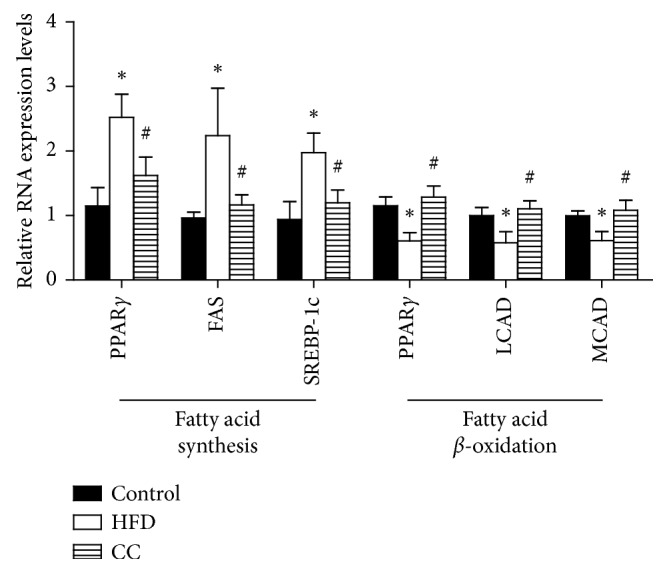
Effects of compound C on lipid metabolism-related genes. Fatty acid synthesis- and oxidation-related gene expression levels were detected by real-time PCR. All data are presented as mean ± SEM. ^*∗*^*p* < 0.05, compared with the HFD group (*n* = 5/group). Control = normal control; HFD = high-fat diet; CC = compound C. ^*∗*^, #<0.05 compared with CC group (*n* = 5/each group).

We next explored the functional role of compound C in hepatic steatosis, the most prominent characteristic of NAFLD development. Regarding liver pathologies, the H&E analysis showed the HFD-induced exacerbation in hepatic lipid accumulation and inflammation, when compared with normal controls. However, the compound C injection for 4 weeks markedly ameliorates histological parameters according to the NAFLD activity score, when compared with mice in the HFD group (Figure 3). In addition, measurement of fatty acid metabolism-related genes showed that compound C significantly decreased the fatty acid synthesis, while increased the fatty acid oxidation (Figure 4). Together, these observations demonstrated that compound C ameliorates HFD-induced hepatic steatosis, suggesting the potential application of compound C for NAFLD treatment.

### 3.3. Compound C Inhibits Proinflammatory Gene Expression in HFD-Induced Liver Injury

**Figure 5:**
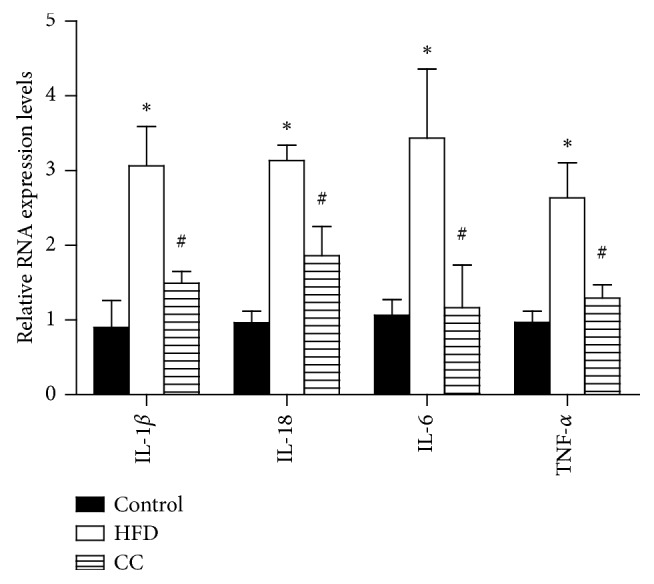
Effects of compound C on proinflammatory gene expression in HFD-induced hepatic steatosis. IL-1*β*, IL-18, IL-6, and TNF-*α* expressions were detected by real-time PCR. All data are presented as mean ± SEM. ^*∗*^*p* < 0.05, compared with the HFD group. Control = normal control; HFD = high-fat diet; CC = compound C. ^*∗*^, #<0.05 compared with CC group (*n* = 5/each group).

Because inflammation has been recognized as a decisive step in NAFLD development and progression, we next evaluated the influence of compound C on crucial proinflammatory genes involved in NAFLD. The transcriptional analysis revealed that the compound C administration significantly inhibited the mRNA expression level of proinflammatory markers in liver tissue, including IL-1*β*, IL-18, IL-6, and TNF-*α*, which have been proposed to play a vital role in the development of steatohepatitis, when compared with mice in the HFD group (Figure 5). Consistent with these gene expression profiles, the inhibitory effect of compound C on these cytokines was further confirmed in LPS plus palmitate-stimulated THP-1 cells (data not shown). These findings suggest that compound C represses inflammatory responses during HFD-induced NAFLD development.

### 3.4. Compound C Suppresses NLRP3 Inflammasome Activation in HFD-Induced Hepatic Steatosis

**Figure 6:**
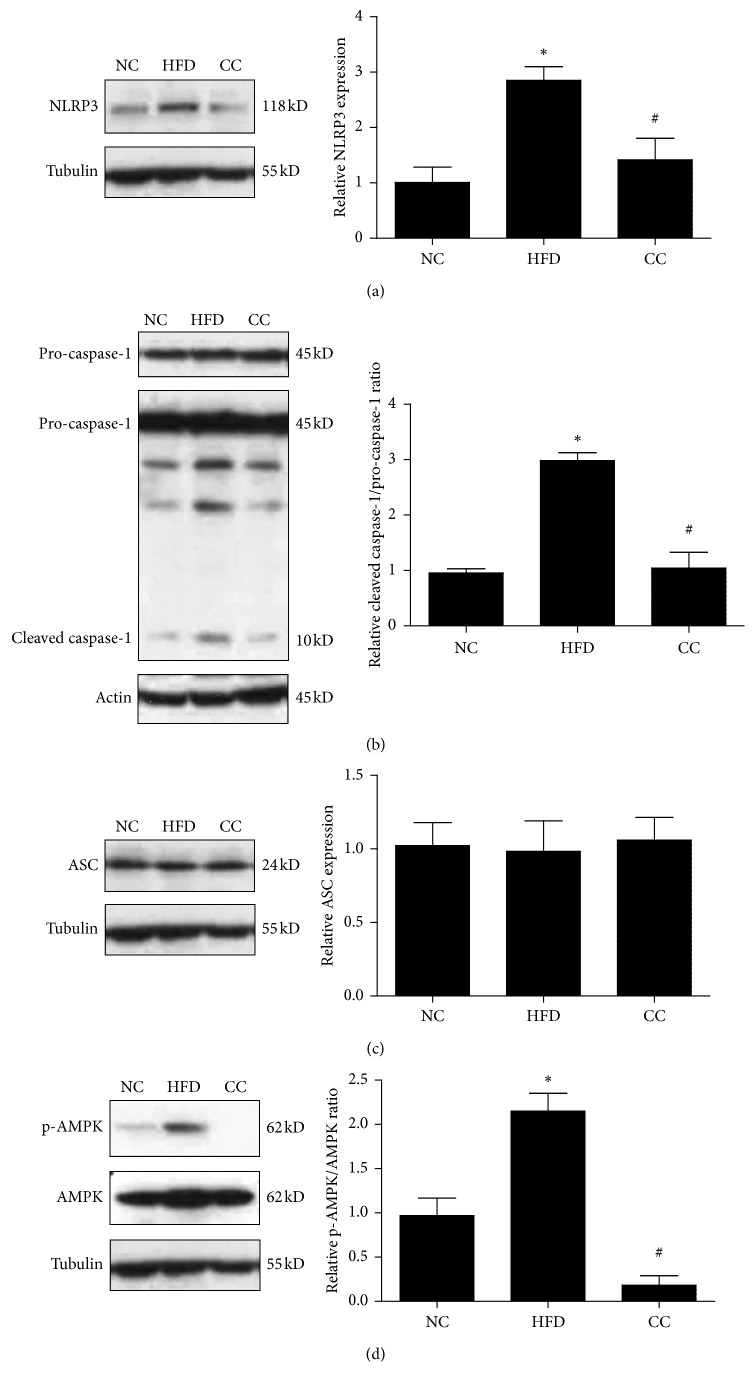
Effects of compound C on NLRP3 inflammasome expression in HFD-induced hepatic steatosis. (a) The compound C group expressed lower levels of NLRP3 compared to the HFD group. (b) The level of cleaved caspase-1 was lower in the compound C group than in the HFD group. (c) Compound C has no effect on ASC expression. All data are presented as mean ± SEM. ^*∗*^*p* < 0.05, compared with the normal chow group. ^#^*p* < 0.05, compared with the HFD group (*n* = 5/each group). NC = normal control; HFD = high-fat diet; CC = compound C. (d) Compound C-treated group expressed lower level of p‐AMPK than HFD‐treated group, but had no effect on AMPK levels.

As inflammation in the liver is believed to be the compelling feature in NAFLD development, the inhibitory effect of compound C on proinflammatory mediators (i.e., IL-1*β* and IL-18) encouraged us to further investigate the underlying mechanisms. Accumulating evidence suggests that NLRP3 inflammasome plays a pivotal role in the pathogenesis of NAFLD/NASH and controls the maturation and release of IL-1*β* and IL-18 [14, 15]. Therefore, we further investigated whether compound C could regulate NLRP3 inflammasome, which is composed of three components: NLRP3, the adaptor protein ASC (apoptosis-associated speck-like protein containing a caspase-recruitment domain), and pro-caspase-1. Notably, in the liver tissue of HFD-fed mice, the expressions of NLRP3 inflammasome-associated proteins (NLRP3 and cleaved caspase-1) were significantly higher than those in the NC group with no significant difference of ASC protein expression (Figure 6). However, in the compound C group, we clearly observed that compound C led to significant reductions in expressions of NLRP3 inflammasome-associated proteins (NLRP3 and cleaved caspase-1), indicating that NLRP3 inflammasome inhibition is one of the main mechanisms through which compound C exerts its effect in attenuating NAFLD.

### 3.5. Compound C Enhances Antioxidative Stress and Reduces p-AMPK/AMPK Ratio Induced by PA

**Figure 7:**
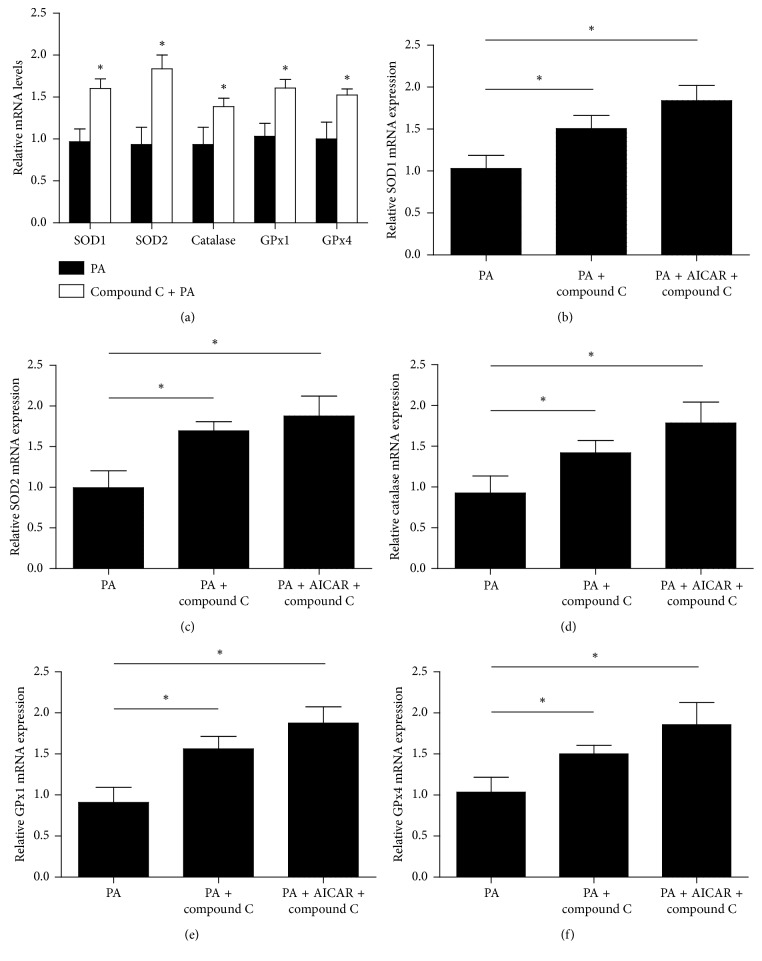
Compound C enhances mRNA levels of antioxidative stress enzymes (a). HepG2 cells were pretreated with PBS, compound C (25 *μ*M), or compound C (25 *μ*M) plus AICAR (500 *μ*M) for 1 h and then treated with PA (0.5 mM) for 3 h. The cells were collected, and mRNA levels of SOD1 (b), SOD2 (c), catalase (d), GPx1 (e), and GPx4 (f) were determined by quantitative real-time PCR. ^*∗*^*p* < 0.05.

**Figure 8:**
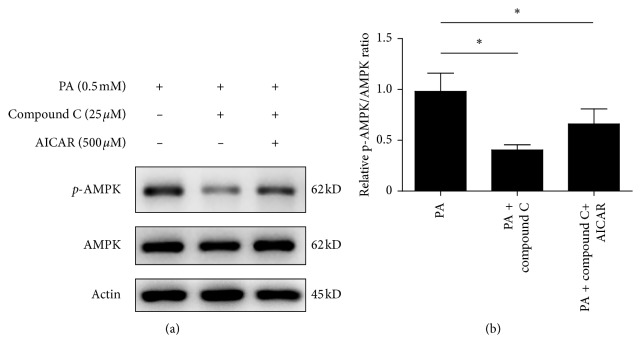
Compound C reduces the p-AMPK/AMPK ratio. HepG2 cells were pretreated with PBS (the left line), compound C (25 *μ*M) (the middle line), or compound C (25 *μ*M) plus AICAR (500 *μ*M) (the right line) for 1 h and then treated with PA (0.5 mM) for 3 h. Cell lysates (30 *μ*g of total protein) were subjected to western blot analyses for p-AMPK, AMPK, and actin. Each blot was repeated 3 times. All data are presented as mean ± SEM. ^*∗*^*p* < 0.05, compared with cells treated with PA.

The HepG2 cell line was originally derived from a hepatocellular carcinoma biopsy and synthesized nearly all human plasma proteins. HepG2 cell culture has been widely used to investigate the oxidative injury of hepatocytes. Cells treated with palmitic acid (PA) for 3 h and mRNA of SOD1, SOD2, catalase, GPx1, and GPx4 were determined. Pretreatment with compound C significantly enhanced the mRNA levels of these antioxidative stress enzymes (*p* < 0.05) (Figure 7(a)). We further pretreated HepG2 cells with compound C plus AICAR for 1 h and then treated with PA for 1 h. The mRNA levels of SOD1, SOD2, catalase, GPx1, and GPx4 in cells co-pretreated with compound C and AICAR were the highest (*p* < 0.05) (Figures 7(b)–7(f)). The results suggest that compound C enhances the mRNA levels of antioxidative stress enzymes, which are stimulated by PA. AICAR does not block the effect of compound C but coenhances the effects on the antioxidative stress enzymes. HepG2 cells were pretreated with PBS, compound C, and compound C plus AICAR for 1 h and then treated with PA for 3 h. Cell lysates were subjected to western blot analyses. Compound C reduced the p-AMPK/AMPK ratio when compared with cells pretreated with PBS (*p* < 0.05). AICAR slightly revised the compound C-caused reduction of the p-AMPK/AMPK ratio (*p* < 0.05) when compared with cells pretreated with PBS (Figure 8).

## 4. Discussion

As one of the most prevalent chronic liver diseases worldwide, NAFLD is a progressive and pathological condition, which currently has no effective treatment strategies. We report here that compound C, an inhibitor of AMPK, exerted potent hepatoprotective functions in HFD-induced NAFLD mice. Compound C suppresses the body-weight gain and insulin resistance, inhibits hepatic steatosis, and regulates lipid metabolism-related genes. As the NAFLD development is a chronic inflammation, we have further demonstrated that compound C also downregulates the essential proinflammatory genes. Furthermore, compound C inhibits NLRP3 inflammasome activation, which plays a key role in the development and progression of NAFLD. Our data suggest that compound C might be a potentially novel therapeutic strategy for the treatment of NAFLD-related metabolic disorders.

Hepatic lipid accumulation accompanied by changes in lipid metabolism is a hallmark of NAFLD, which mediates hepatocellular apoptosis due to palmitate-induced lipotoxicity [15]. The underlying mechanism leading to excessive lipid accumulation in NAFLD can be attributed to an enhanced synthesis of fatty acids and inhibition of fatty acid oxidation. In this study, compound C significantly reduces hepatic steatosis and reduces the activity of the lipogenic genes, including peroxisome proliferator‐activated receptor gamma (PPARγ), fatty acid synthase (FAS), and sterol regulatory element-binding protein 1c (SREBP1c), which are critical transcriptional regulators for the target genes involved in lipid synthesis [16]. Moreover, the impairment of the fatty acid oxidation system represents another important mechanism in hepatic lipid accumulation, in which PPAR*α*, long-chain acyl-CoA dehydrogenase (LCAD) scale, and medium-chain acyl-CoA dehydrogenase (MCAD) are involved [17]. We have found a considerable increase in the gene expression of PPAR*α*, LCADL, and MCAD in compound C-treated NFD mice, suggesting that compound C could enhance the fatty acid oxidation.

The low-grade chronic inflammation is another remarkable feature in obesity and obesity-related metabolic disorders, such as atherosclerosis, type 2 diabetes mellitus, and NAFLD [18]. As a critical pathogenesis of NAFLD, the inflammation is a link between insulin resistance and hepatic lipid accumulation. In this aspect, it is interesting to note that compound C reduces the inflammatory gene expression in the liver, including IL-1*β*, IL-18, IL-6, and TNF-*α*. Consistent with our findings, Lee et al. demonstrated that compound C pretreatment resulted in the expression of proinflammatory cytokines, such as TNF-*α*, IL-6, IL-1*β*, and MCP, in RAW264.7 cells stimulated with LPS, and unexpectedly found that compound C inhibited the inflammatory response [13]. IL-1*β* is an important proinflammatory cytokine that drives the pathogenesis of liver inflammation, hepatic steatosis, liver fibrosis [19, 20], and insulin resistance by impairing insulin signaling in the liver, muscle, and adipose tissue [21]. IL-1*β* can also initiate a self-amplifying cytokine network with generation of other inflammatory mediators by activation of IL-1 receptors, indicating that IL-1*β* is prominent and one of the major proinflammatory cytokines. Interleukin-18 (IL-18) is also a proinflammatory cytokine associated with NAFLD. Mitsuyoshi reported that hepatic IL-18 mRNA and serum IL-18 levels were significantly higher in NAFLD patients than those in controls, which correlated significantly with hepatic steatosis [22]. Flisiak-Jackiewicz et al. also showed that elevated serum IL-18 concentration and its correlation with hepatocyte injury, systemic inflammation, and degree of liver steatosis support an important role in NAFLD pathomechanism [23]. Therefore, IL-18 can be considered to play a functional role in predicting advanced liver steatosis and fatty liver in obese patients. The inflammatory alleviation of compound C in NAFLD, especially the inhibition effect on IL-1*β* and IL-18, led us to further explore the potential target.

Recently, NLRP3 inflammasome is reported to be an important contributor to the development of NAFLD [24–26]. NLRP3 inflammasome can be activated by metabolic danger signals, such as free fatty acids, oxidized LDL, cholesterol crystals, and uric acid [27–30]. In response to the danger signals, NLRP3 recruits ASC together with pro-caspase-1 to form the complex “NLRP3 inflammasome,” then leads to activation of cleaved caspase-1, and subsequently cleaves pro-IL-1*β* and pro-IL-18 into bioactive forms IL-1*β* and IL-18. Thus, the processing of IL-1*β* and IL-18 is tightly regulated and controlled by NLRP3 inflammasome. Given the inhibition effect of compound C on IL-1*β* and IL-18, it is reasonable to hypothesize that compound C may regulate NLRP3 inflammasome, which is the upstream of IL-1*β* and IL-18. Along this line, we have found a considerable increase in NALP3 and active caspase-1 expression in HFD mice, which was downregulated by compound C to levels of control mice. Despite there were no significant changes of ASC expression, NLRP3 and caspase-1 are more important and functional components of NLRP3 inflammasome. Further studies are required to elucidate how compound C functioned on NLRP3 inflammasome.

Accordingly, an earlier work reported that compound C significantly inhibited adipogenic differentiation of 3T3-L1 cells in a dose-dependent manner and caused downregulated expression levels of early adipogenic transcription factors [31]. The authors proposed that their results suggest compound C might serve as a useful molecule in adipogenesis and as a potential lead compound for the treatment of obesity and obesity-related disorders. Furthermore, it was reported that intracerebroventricular or intraperitoneal injection of compound C in mice reduced food intake, which may subsequently cause weight loss [32, 33]. These observations agree with those of our study. Therefore, it is likely that compound C could improve metabolic diseases probably through its anti-inflammatory property.

Compound C belongs to pyrazolopyrimidine-like protein kinase inhibitors. The structural group of compound C for inhibiting inflammation and inflammasome might be the group apart from inhibition of AMPK. It is not clear whether compound C acts directly on the inflammasome, but compound C inhibits oxidative stress ROS [6]. Compound C is also a competing reversible ATP binder [3]. Because NLRP3 protein contains an ATP-binding region, compound C may also be a direct inhibitor of NLRP3. We have also found that compound C enhances the mRNA levels of antioxidative stress enzymes in human hepatoma HepG2 cells. The effect could be enhanced by AICAR.

In conclusion, we have shown that compound C has excellent anti-inflammatory characteristics. The action of compound C largely depends on direct inhibition of NLRP3 inflammasome, leading to ameliorated inflammation, insulin resistance, and hepatic accumulation. Compound C also enhances the mRNA levels of antioxidative stress enzymes and reduces the p-AMPK/AMPK ratio, which are stimulated by PA. Taken together, our study supports primary evidence suggesting that compound C is a potent NAFLD suppressor and an attractive therapeutic target for hepatic steatosis and related metabolic disorders.

## Data Availability

The authors retain the original data and FACS data and graphs, which are available upon request to the corresponding author at email id 2114386136@qq.com.

## Abbreviations

LCAD: Long-chain acyl-CoA dehydrogenase
MCAD: Medium-chain acyl-CoA dehydrogenase
ASC: Caspase-recruitment domain
FAS: Fatty acid synthase
GPx: Glutathione peroxidase
PPAR*α*: Peroxisome proliferator-activated receptor alpha
PPAR*γ*: Peroxisome proliferator-activated receptor gamma
SOD1: Zinc superoxide dismutase
SOD2: Manganese superoxide dismutase
SREBP-1c: Sterol regulatory element-binding transcription factor 1c.
